# Regularity of Center of Pressure Trajectories in Expert Gymnasts during Bipedal Closed-Eyes Quiet Standing

**DOI:** 10.3389/fnhum.2017.00317

**Published:** 2017-06-20

**Authors:** Brice Isableu, Petra Hlavackova, Bruno Diot, Nicolas Vuillerme

**Affiliations:** ^1^Aix Marseille Univ, PSYCLEAix-en-Provence, France; ^2^Équipe d’Accueil Autonomy, Gerontology, E-health, Imaging & Society, Université Grenoble-AlpesGrenoble, France; ^3^Grenoble Alpes University HospitalGrenoble, France; ^4^Informatique de SécuritéMontceau-les-Mines, France; ^5^Institut Universitaire de FranceParis, France

**Keywords:** balance, entropy

## Abstract

We compared postural control of expert gymnasts (G) to that of non-gymnasts (NG) during bipedal closed-eyes quiet standing using conventional and nonlinear dynamical measures of center of foot pressure (COP) trajectories. Earlier findings based on COP classical variables showed that gymnasts exhibited a better control of postural balance but only in demanding stances. We examined whether the effect of expertise in Gymnastic can be uncovered in less demanding stances, from the analysis of the dynamic patterns of COP trajectories. Three dependent variables were computed to describe the subject’s postural behavior: the variability of COP displacements (A_CoP_), the variability of the COP velocities (V_CoP_) and the sample entropy of COP (SEn_CoP_) to quantify COP regularity (i.e., predictability). Conventional analysis of COP trajectories showed that NG and G exhibited similar amount and control of postural sway, as indicated by similar A_CoP_ and V_CoP_ values observed in NG and G, respectively. These results suggest that the specialized balance training received by G may not transfer to less challenging balance conditions such as the bipedal eyes-closed stance condition used in the present experiment. Interestingly, nonlinear dynamical analysis of COP trajectories regarding COP regularity showed that G exhibited more irregular COP fluctuations relative to NG, as indicated by the higher SEn_CoP_ values observed for the G than for the NG. The present results showed that a finer-grained analysis of the dynamic patterns of the COP displacements is required to uncover an effect of gymnastic expertise on postural control in nondemanding postural stance. The present findings shed light on the surplus value in the nonlinear dynamical analysis of COP trajectories to gain further insight into the mechanisms involved in the control of bipedal posture.

## Introduction

Posture can be defined as the spatial organization of the body segments (e.g., Winter, 1995). Postural regulation is a complex skill that requires coordinating and controlling subtle rotational movements of hundreds of joints by means of several hundreds of muscles to maintain the center of mass within the base of support. The multisensory consequences of the kinematics and kinetic variations patterns of postural movements, i.e., the dynamics of postural balance, would be informative of the direction of balance (DOB, Riccio et al., 1992) and preferred modes of spatial referencing (Streepey et al., 2007a,b; Isableu et al., 2010; Slaboda and Keshner, 2012). To maintain a bipedal posture stable, central processing factors are known to play a major role insofar as the central nervous system has to process information from various sensory cues (visual, somesthetic and vestibular), and weight them in proportion to their reliabilities (Oie et al., 2002). Analysis of the center of pressure (COP) in various upright stance tasks is widely used to characterize postural control and to understand the underlying motor control mechanisms during challenging experimental conditions. Force platform is typically used to assess the location and the dynamics of the COP. COP dynamics are likely due to complex control process associated with the maintenance of postural control, as well as the inherent noise within the human neuromotor system. COP is widely used to assess the health of the postural control system, but also to learn about the effect of athletic expertise (Lion et al., 2009; Herpin et al., 2010; Paillard et al., 2011; Zemková, 2014a,b). Previous studies investigated postural control during quiet standing in expert gymnasts (G), a sport requiring high balance abilities (Vuillerme et al., 2001a,b; Asseman et al., 2004, 2008; Vuillerme and Nougier, 2004; Gautier et al., 2008). Interestingly, these studies reported no significant difference between gymnasts and non-gymnasts (NG) under relatively non-challenging conditions (bipedal eyes-open posture). Authors suggested that expertise in gymnastics only has an effect on the control of specific postures for which the practice is specifically related to (see also, Henry, 1968; Schmidt and Young, 1987). However, standing posture during an eyes-closed bipedal standing task is known, as a test condition that increases reliance on vestibular (and proprioceptive) input (Rougier, 2003; Isableu and Vuillerme, 2006; Isableu et al., 2010), but also to require attention demands in gymnast and NG (Vuillerme and Nougier, 2004). At this point, however, the common observation from these studies is that the use of conventional measures of the center of foot pressure (COP; e.g., COP surface area, COP velocity) to quantify postural control in expert gymnasts may have yielded an incomplete picture of postural control in expert gymnasts (Asseman et al., 2004; Vuillerme and Nougier, 2004). Analyses carried out on nonlinear dynamic features of the COP revealed that variability in the motor output is not randomness but structured. Further insight into the underlying dynamics of bipedal eyes-closed postural control in expert gymnasts could be obtained through the recourse to nonlinear dynamical analysis of the COP regarding its regularity (i.e., predictability) using sample entropy measures (SEn_CoP_; Borg and Laxåback, 2010). Interestingly, a more irregular COP trajectory, as assessed by higher SEn_CoP_, has been suggested to be associated with more automaticity and has been proposed to be viewed as a reduction of the amount of attention invested in the control of posture (e.g., Roerdink et al., 2006, 2009, 2011; Donker et al., 2007; Stins et al., 2009a,b; Manor et al., 2013; Bieć et al., 2014; Wayne et al., 2014).

The present experiment was designed to address the relationship between attention invested in posture and COP regularity by comparing postural control of expert gymnasts to that of NG during bipedal eyes-closed standing using both conventional and nonlinear dynamical measures of the COP trajectories. The two underlying hypotheses are: (A) The extensive postural control training that gymnasts receive over the years changes the requirements on their postural control system in such a way that for the same balance task they require less attentional resources than NG; and (B) If more attentional resources are invested in a postural control task, then the COP movement becomes more regular, if, on the other hand, the postural task is controlled more by automated processes, then the COP movement characteristics become more irregular or complex (e.g., Roerdink et al., 2006, 2009, 2011; Donker et al., 2007; Stins et al., 2009a,b; Manor et al., 2013; Wayne et al., 2014).

From these two hypotheses, the following prediction can be derived: if both hypotheses are correct, then the sample entropy, a measure of irregularity of a time series, calculated for the COP of gymnasts should be higher than the SEn_CoP_ of NG. Hence, the purpose of the current study was to test the two hypotheses by confirming or refuting this prediction.

As a result, taking into account the above-mentioned results (Vuillerme et al., 2001a,b; Asseman et al., 2004, 2008; Vuillerme and Nougier, 2004), no significant difference between conventional measures of the COP measured in gymnasts and those measured in NG were expected. On the other hand, and more *originally*, considering: (1) the decreased attentional demand required for regulating postural sway during quiet standing previously reported in gymnasts relative to NG using a dual-task paradigm (Vuillerme and Nougier, 2004); and (2) the proposed relationship between the amount of attention invested in posture and COP regularity (e.g., Roerdink et al., 2006, 2009, 2011; Donker et al., 2007; Stins et al., 2009a,b; Manor et al., 2013; Wayne et al., 2014), gymnasts were expected to exhibit more irregular COP trajectories, operationalized with higher SEn_CoP_, values, than NG.

## Materials and Methods

### Subjects

Two groups of athletes voluntarily participated in the experiment. They were naïve as to the purpose of the study. This study was carried out in accordance with the recommendations of the local Ethics Committee with written informed consent from all subjects. All subjects gave written informed consent to the experimental procedure in accordance with the Declaration of Helsinki. The protocol was approved by the local Ethics Committee.

The group of expert gymnasts (G) consisted of 10 males having more than 10 years of experience (8 h/week) in gymnastics competition at the regional level or higher. Females were not considered in this study to remove potential bias due to: (i) known influence of anthropometric factors and gender on postural balance in adults (Chiari et al., 2002; Farenc et al., 2003; Alonso et al., 2012); but also because (ii) mechanical, and skeletal differences known to produce different neuromuscular control of the knee joint (Shultz and Perrin, 1999) on body sway resulting in a different postural response (Schmitz et al., 2007; Ku et al., 2012) to sensory alteration (Raffi et al., 2014); and (iii) sensory integration difference with men favoring visual dependency (Raffi et al., 2014; Persiani et al., 2015). Since our findings may originate simply from the practice of sports in general, gymnasts’ performance was compared to the performance of a control group composed of 10 NG males who were also experts in sport (soccer, handball, or tennis). We also adjusted the composition of the two groups such that there was no significant difference either in age, weight and height (Table 1) because body properties have been demonstrated to be determinant for postural task (Chiari et al., 2002; Ruhe et al., 2010).

**Table 1:** Age, weight, height of Non-gymnasts (NG) and Gymnasts (G) groups.

	Non gymnasts (*n* = 10)	Gymnasts (*n* = 10)	*T*-test (*P* < 0.05)
Age (years)	22.0 ± 1.3	21.9 ± 1.0	Ns
Weight (kg)	68.3 ± 2.9	67.5 ± 2.0	Ns
Height (cm)	173.9 ± 3.3	170.9 ± 3.1	Ns

*Values are means and standard deviation (±); Ns = non-significant difference between the two groups*.

### Experimental Procedure

Subjects stood barefoot on the force platform (Dynatronic, France) in a standardized position (feet abducted at 30°, heels separated by 3 cm), their arms hanging loosely by their sides with eyes closed. This closed eyes condition has been chosen to avoid visual information interfering with the control of bipedal posture. Indeed, given the crucial role of visual information (for a review, see Redfern et al., 2001), earlier studies provided evidence that the eyes-closed condition in evaluating postural control helps to improve the discrimination between healthy people (see Isableu and Vuillerme, 2006; Isableu et al., 2010), and patients with sensory (e.g., vestibular; Horak et al., 1990; Allum et al., 2001), somesthetic (Oppenheim et al., 1999; Nardone et al., 2001) or sensory-motor (Marigold and Eng, 2006; Blaszczyk et al., 2007) impairments. In fact, the availability of visual information allows individuals to compensate for their postural deficits (for a review, see Redfern et al., 2001) limiting the use of the eyes-open condition as a normative based clinical protocol for objective evaluation of postural control, particularly if vestibular or somesthetic functions have to be assessed (Hlavačka, 2003). As a consequence, the eyes-open condition was not measured in this study. Subject’s task was to stand as still as possible during the trial.

Three 30 s trials were performed. Rest periods of 60 s were provided between successive trials during which subjects were allowed to sit down.

Data were recorded at a sampling frequency of 40 Hz which is large enough for capturing the physiological content of the postural signal localized below 5 Hz and which is equal or larger than the sampling frequency used in others studies (Cavanaugh et al., 2007; Ramdani et al., 2009, 2011; Borg and Laxåback, 2010; Rhea et al., 2011).

Collected data were protected by the MedSafe technology by the IDS Company (Montceau-les-Mines, France). IDS Company is an approved hosting provider in personal health data by the French Ministry for Social Affairs and Health.

### Data Analysis

The anteroposterior and mediolateral COP time series were centered on zero mean before constructing the resultant distance COP time series. Specifically, the resultant distance is the vector distance from the center of the posturogram to each point in the posturogram and hence it is not sensitive to the orientation of the base of support on force platform (Prieto et al., 1996).

Three dependent variables computed from the resultant distance COP were used to describe the subject’s postural behavior using a similar methodology as recently proposed by Roerdink et al. (2009, 2011). The “amount of sway” and the “sway control” were quantified using two conventional, scale-dependent measures (see Prieto et al., 1996; Donker et al., 2007):

(1) the variability of COP displacements (A_CoP_ in mm, expressed as the root mean square of the COP time series),
(2) the variability of the COP velocities (V_CoP_ in mm/s, expressed as the root mean square of the COP velocities time series);

To examine the dynamical structure of COP trajectories and index its regularity independent of the size or scale. To this end, the RD time series was normalized to zero mean and unit variance resultant distance by subtracting its mean from this time series and dividing it by its standard deviation. Subsequently,

(3) the sample entropy of COP (SEn_CoP_, dimensionless) was quantified for RD distance time series (Roerdink et al., 2009, 2011). Note that sample entropy was not calculated for the resultant distance differenced time series as suggested by Ramdani et al. (2009) to eliminate the inherent non-stationary nature of COP trajectories. Indeed, Roerdink et al. (2011) showed that it yields similar results. Algorithms of Lake and colleagues (Lake et al., 2002; Richman et al., 2004) were used to estimate corresponding sample entropy values. The sample entropy in a set of data points is the negative natural logarithm of the conditional probability (CP = A/B) that a sequence of data points with length *N*, having repeated itself within a tolerance *r* for *m* points, will also repeat itself for *m* + 1 points, without allowing self-matches (Richman and Moorman, 2000; Lake et al., 2002). Accordingly, B represents the total number of matches of length m while A represents the subset of B that also matches for *m* + 1. Sample entropy thus follows from −log (A/B), with a low sample entropy value arising from a high probability of repeated template sequence in the data. In this context, entropy is the rate of generation of new information and the lower the entropy, the greater the regularity (predictability) of the time series in question.

The reliability of the sample entropy estimation depends on the parameter choice of *m* and *r*. Sample entropy is best estimated with *m* as large and *r* as small as possible (Roerdink et al., 2009, 2011). Lake et al. (2002) introduced a statistical criterion to optimize the parameter choice, which is based on the maximum of the relative error of sample entropy and the conditional probability estimates. This metric simultaneously penalizes the conditional probability near 0 and near 1 (Lake et al., 2002) and represents the tradeoff between accuracy and discriminative capability. The criterion was set to be no higher than 0.05, implying that the 95% confidence interval of the sample entropy estimate is maximally 10% of its value (Lake et al., 2002). Ramdani et al. (2009, 2011) recently proposed a practical graphical method based on a convergence criterion to optimize the choice of the parameter values. This optimization procedure was notably used by Roerdink et al. (2011) who found (*m* = 3, *r* = 0.05) to be the optimal couple (see Rhea et al., 2011; Hansen et al., 2017). This result is comparable to other couple of parameters previously obtained from the original optimization procedure proposed by Lake et al. (2002) for the resultant distance times series too (Donker et al., 2007; Roerdink et al., 2009). Therefore, this couple was also used in this study to perform the calculation of sample entropy (Hansen et al., 2017).

### Statistical Analysis

The mean of ACoP, VCoP and SEnCoP values obtained for each of three trials were averaged for statistical analysis. COP data being normally distributed, A_CoP_, V_CoP_ and SEn_CoP_ obtained in the NG group were compared with those obtained in the G group using *t*-tests for independent measures. Statistical analyses were performed using Statistica 10. Level of significance was set at 0.05.

## Results

**Figure 1:**
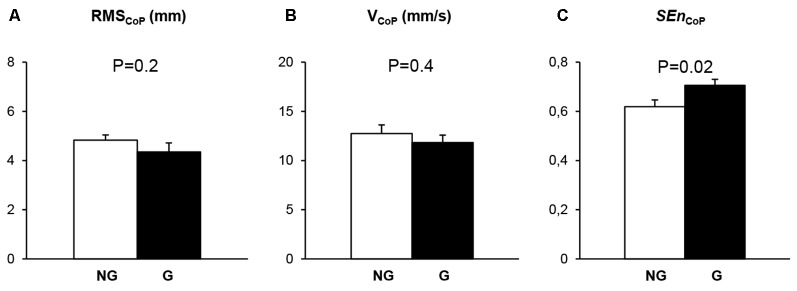
Mean and standard error of mean of the variability of the displacements (A_CoP_; **A**), the velocity (V_CoP_; **B**) and the regularity (*SEn*_CoP_; **C**) of the center of foot pressure (COP) trajectories obtained in the two groups of Non gymnasts (NG; *white bars*) and Gymnasts (G; *black bars*). The *P* values for comparisons between postural parameters computed from the NG and those computed from the G are reported.

Statistical difference between the NG and the G was observed neither for the A_CoP_ (*t* = −1.20, *P* = 0.25, Figure 1A) nor for the V_CoP_ (*t* = −0.83, *P* = 0.42, Figure 1B). Conversely, SEn_CoP_ was significantly higher in G than in NG (*t* = −2.48, *P* = 0.023, Figure 1C).

## Discussion

Regarding the conventional posturographic analysis of COP trajectories, our results showed that NG and G exhibited similar amount and control of postural sway, as indicated by similar A_CoP_ (Figure 1A) and V_CoP_ values (Figure 1B) observed in NG and G, respectively. These results confirmed previous observations (Vuillerme et al., 2001a,b; Asseman et al., 2004, 2008) supporting the general idea according to which, the postural control capacities are specific to the training program and the requirements of each discipline. The specialized balance training received by gymnasts may not transfer to less challenging balance conditions such as the bipedal eyes-closed stance condition used in the present experiment (see also, Henry, 1968; Schmidt and Young, 1987). However, Vuillerme and Nougier (2004), using a stimulus-responses reaction time paradigm to assess attentional investment, reported a smaller attentional involvement in balance control for expert gymnasts than for NG. Interestingly, in this study, the main effect of expertise assessed via classical COP variables was not significant. These results suggested that some variables used in conventional posturographic analysis of COP trajectories did not capture the amount of attention invested to control postural balance. One reason is that most variables used in conventional posturographic analysis of COP trajectories are* a priori* more suited to capture linear stationary processes (i.e., additive phenomenon) hidden in signal fluctuations (Wayne et al., 2014; Gow et al., 2015), and as a consequence fail to capture complex central interaction that result from the combination of both additive and multiplicative processes (Huang et al., 2016). The results mentioned above suggest that attentional mechanisms likely involve complex neural interaction and nonlinear processes (i.e., a mixture of additive and multiplicative phenomenon). Hence, attentional-based interactions and the amount of attentional investment in postural control seem better captured in the COP fluctuations by using nonlinear (multiplicative) variables.

Regarding the nonlinear dynamical posturographic analysis of COP trajectories regarding COP regularity, our results showed indeed that G exhibited more irregular COP fluctuations relative to NG, as indicated by the higher SEn_CoP_ values observed for the G than for the NG (Figure 1C). This result shows that nonlinear variables (SEn_CoP_) are more appropriate to capture nonlinear multiplicative processes in the COP signal. Following the proposed relation between COP regularity and the amount of attention invested in the control of posture (e.g., Stins et al., 2009a), these results and ours suggest less attentional investment, i.e., a more fully automatized form of balance, in experts in sports requiring fine postural control (i.e., dancers and gymnasts) than controls. Our results are in accordance with those of Vuillerme and Nougier (2004) who, using a stimulus-responses reaction time paradigm to operationalize attentional investment, reported a smaller attentional involvement in balance control for expert gymnasts than for NG. Although to the best of our knowledge, no previous study has assessed regularity of COP trajectories in expert gymnasts during bipedal eyes-closed quiet standing, our observation is in line with a recent result obtained in experts in dance (Stins et al., 2009b), a sport that also require high balance abilities. Stins et al. (2009b) reported higher SEn_CoP_ in preadolescent pre-professional dancers than age-matched non-dancers. An alternative explanation of our findings could be drawn from the Borg and Laxåback’s (2010) study. The higher COP entropy observed in gymnasts relative to nongymnasts suggests they exhibited a more automatic balance control. Within this view, higher COP entropy could indicate that they deployed a more efficient balancing. The efficiency with which postural balance (low COP variability and low attentional investment) is controlled is closely tied to the selection of an appropriate mode of spatial referencing (generally proprioceptive-based; Berthoz, 1991; Paillard, 1991; Kluzik et al., 2005; Streepey et al., 2007b; Isableu et al., 2010, 2011; Mergner, 2010; Slaboda et al., 2011a,b; Brady et al., 2012; Scotto Di Cesare et al., 2015). Several authors showed that these modes of spatial referencing are known to impact the attentional investment (Goodenough et al., 1987; Marendaz et al., 1988; Marendaz, 1989; Bailleux et al., 1990; Yan, 2010; Agathos et al., 2015). Following this rationale, it is likely that with the selection of the adequate frame of reference, attentional investment should decrease, and accounts for the emergence of more irregular (more complex) COP time series (Vuillerme and Nougier, 2004), even in nondemanding stance.

Finally, two main conclusions can be drawn from the differential effect of expertise in gymnastics observed on the conventional (Figures 1A,B) and the nonlinear dynamical measure of the COP trajectory (Figure 1C) during bipedal eyes-closed quiet standing. First, these results suggest that, under mild challenging postural condition such as bipedal eyes-closed stance, postural control in expert gymnasts is *qualitatively*, but not *quantitatively*, different than that of controls. Although the expert population is different, the present findings are in line with those of Manor et al. (2013) and Wayne et al. (2014) on the impact of short- and long-term Tai Chi exercise training. These authors also reported that the effect of Tai Chi on postural control may be better characterized by quantifying its effects on the degree of complexity associated with the system output (i.e., COP dynamics) than by the traditional sway parameters (Manor et al., 2013; Wayne et al., 2014). Indeed, using both standard measures of postural sway and recurrence quantification analysis, these authors (Manor et al., 2013; Wayne et al., 2014) observed that trained ballet dancers exhibited similar variability and amount of postural sway, but more irregular sway and thus complex patterns than physically fit control group. Second, the observation that the balance skills of gymnasts were observed in the dynamic patterns of COP displacements (Figure 1C), but not in the control (Figure 1A) and the amount of postural sway velocity (Figure 1B) shed light on the surplus value in nonlinear dynamical analysis of COP trajectories to gain further insight into the mechanisms involved in the control of bipedal eyes-closed posture. Along these lines, some limitations of our study can be pointed. Nonlinear dynamics features of the COP displacements could have been explored in more depth using Multi-Scale Entropy (MSE), and Multivariate Multi-Scale Entropy (MMSE). These methods are particularly suitable to quantify the degree of regularity or predictability over multiple scales of time (see Costa et al., 2005; Gow et al., 2015). Our analyses were mainly carried out on the original time series. Additional information can be obtained from the analysis of the decremented time series (which removes the long-term correlated components from the original time series and represent short-term complexity). Hansen et al. (2017), showed that MMSE analysis performed on the decremented time series is particularly suitable to detect signal divergence faster and can, therefore, be considered more suitable for complexity detection. Further experiments are currently performed to assess the relationship between variation of attentional ressources allocated to control potural balance and complexity of the COP at different scales, but also whether and how characteristics other than sportive expertise, such as anthropometry, neuromuscular state or preferred modes of spatial frames of reference (Streepey et al., 2007b; Isableu et al., 2010, 2011; Slaboda and Keshner, 2012; Agathos et al., 2015), that have been shown to affect balance control, could also modify the dynamical structure of the COP trajectories in terms of their regularity and complexity at different scales and frequency bands (by decomposing the original time series into intrinsic mode functions via empirical mode decomposition techniques (Costa et al., 2005; see Wei et al., 2012; Shih et al., 2015; Hansen et al., 2017).

## Author Contributions

BI, PH, BD and NV conceived and designed the experiment, performed the experiment, analyzed the data, contributed reagents/materials/analysis tools, wrote the article, prepared figures and/or tables, reviewed drafts of the article.

## Conflict of Interest Statement

The authors declare that the research was conducted in the absence of any commercial or financial relationships that could be construed as a potential conflict of interest.
